# The Emulation and Adaptation of a Global Model of Clinical Practice Guidelines on Chronic Heart Failure in BRICS Countries: A Comparative Study

**DOI:** 10.3390/ijerph17051735

**Published:** 2020-03-06

**Authors:** Tao Liu, Benjamin Quasinowski, André Soares

**Affiliations:** 1 School of Public Affairs, Zhejiang University, Hangzhou 310058, China; 2 Institute for Sociology, University of Duisburg-Essen, 47057 Duisburg, Germany; andre.araujo-soares@stud.uni-due.de; 3 Institute of East Asian Studies, University of Duisburg-Essen, 47057 Duisburg, Germany

**Keywords:** BRICS, chronic heart failure, clinical practice guidelines, world-polity, neoinstitutionalism, global health, isomorphism

## Abstract

Whilst knowledge about diseases is universal, access to health care is not equally distributed. During the last decade, the countries of BRICS (Brazil, Russia, India, China, South Africa) have become important actors on the global health scene, pushing for universal, affordable, and more equal access to health care. Although non-communicable diseases place a significant burden on all populations and health systems, low- and middle-income countries (LMIC), such as BRICS, have been affected particularly hard. Approximately 80 percent of worldwide deaths from non-communicable diseases occur in LMIC. We examined if guidelines concerning chronic heart failure from BRICS countries are influenced by global scripts and if these guidelines have converged or diverged in an inter-state context. Our analysis shows that guidelines on heart failure published in BRICS predominantly rely on models initially formulated by European or American cardiological organisations. Guidelines from BRICS deviate from these models to some extent, in particular with regard to specific epidemiological conditions. Except for the Indian guideline, they do not, however, extensively engage with BRICS-specific aspects of costs, access to and affordability of health care services. We interpret these results through the lens of sociological theories on globalisation. Consistent with neoinstitutionalism, recommendations for clinical practice guidelines have spread in BRICS countries in a rather isomorphic fashion. Notwithstanding, some local medical traditions have also been included into these guidelines through localised adaptation and variation.

## 1. Introduction

### 1.1. Background

Since clinical practice guidelines (CPGs) ‘summarize and evaluate all available evidence on a particular issue at the time of the writing process’ [1] (p. 2134) and human biology is commonly considered to be essentially the same around the world [2], recommendations based on the best available biomedical evidence can be applied in any country or health system of the world. Or can they? In fact, the worldwide applicability of CPGs that are developed in and for specific regions and health systems has become the subject of vigorous debates in the global health community [3,4,5]. Many opponents of ‘guideline import’, who are often authors from the Global South, argue that CPGs must be tailor-made for each specific country or health system. Guidelines should not be mere copies from the West [3].

Notwithstanding this debate, one cannot help but notice that there is apparently little variation in CPGs around the world. A cursory glance into any medical field with global reach gives the impression that just a small number of CPGs dominate a particular field. Health organisations all over the world seem to orient their actions and policies to such ‘global scripts’, including models, concepts and methods. This, in turn, apparently leads to cross-national convergence of guidelines.

Such processes of diffusion, transfer and convergence have been described for a plethora of policy fields [6,7,8]. Typically, globally dominant policy models have their origins in the Global North. On the other hand, countries of the Global South are rarely represented in the production side of such powerful models. Nonetheless, these same countries are often eager to integrate the globally dominant models and concepts in their own processes of policy making [6].

In the case of CPGs, however, such considerations have not been confronted with empirical data. Despite the intuition that CPGs are more or less the same everywhere, little is actually known about the degree of convergence between globally dominant CPG models and CPGs being developed in the Global South. Moreover, just as little empirical research has been conducted on the extent to which CPGs from countries of the Global South represent genuinely local(ised) versions.

Therefore, this article examines how a globally dominant CPG model for the diagnosis and treatment of chronic heart failure, with its origins in Europe and the United States, was created, and then adopted, as well as adapted, by health organisations from BRICS (Brazil, Russia, India, China, South Africa). Furthermore, the article demonstrates how CPGs from these countries converge with and partly diverge from the global CPG model as well as with and from one another.

### 1.2. BRICS—A New Actor on the Global Health Scene

The countries of BRICS represent 42.6 percent of the world’s population and its economies account for 23.7 percent of worldwide gross domestic product [9]. Although its sphere of influence is mainly the Global South, over the past decade, BRICS has managed to establish itself as an important actor on the global health scene amidst such clubs as the G7 and the G20. BRICS is well known for its endeavours to foster economic collaboration. A remarkable achievement in this direction was the foundation of the New Development Bank in 2015, intended to provide loan conditions more suitable for many countries repelled by the conditions offered by the IMF or the World Bank. BRICS thus embodies an alternative vision of development, a vision distinct from those predominantly associated with the economies of the Global North [10].

However, BRICS ostensibly has the potential to shift the prevailing paradigms of global health, e.g., with regard to issues of worldwide and inner-country health inequalities [11,12,13]. Since 2011, BRICS health ministers have summited along with the annual World Health Assembly. Their countries share a number of health challenges, as was officially declared in the 2011 Beijing Declaration [14,15]. Consequently, BRICS health ministers have developed a number of genuine ideas and concepts for global health, which decidedly differ from those usually prioritised on the agendas of the G7 and related actors of the Global North. In the years following the Beijing Declaration, BRICS health ministers agreed to address a number of common goals in relation to cost-effective and affordable access to health technologies and medicines, along with universal health coverage and surveillance of health risks [16]. They also agreed on the importance of collaboration and the exchange of knowledge [17].

A series of compliance reports published by the BRICS Information Centre suggests [18] that these declarations of intention are not mere discourse. They regularly engender material infrastructures with perspectives to bring about real changes. For instance, as a consequence of the health ministers’ long-standing engagement against tuberculosis (TB) [19] and in an effort to foster collaborative research and developments in this area, BRICS created the South Africa-based TB Research Network. Although such initiatives were initially focused on the field of infectious diseases, more recently, they have started to include non-communicable diseases (NCDs) as well. This is evident, for example, in the club’s endorsement of the World Health Organisation’s Global Action Plan for Prevention and Control of Non-Communicable Diseases [20]. Furthermore, after the Agenda 2030, with its Sustainable Development Goals, had been adopted by the UN in 2015 [21], BRICS health ministers’ 2016 communiqué directed special attention to the reduction in premature deaths due to NCDs by one third by the year 2030 [22].

In sum, from a sociological perspective, BRICS can be conceived of as an actor that has the power to (re-)shape ‘contexts of inequality’ [23] for large parts of the world’s population. The constant articulation of NCDs on BRICS’s ‘emerging global health agenda’ cannot go unnoticed [11] (p. 15). As the club is continuously emphasising its role as an autonomous actor on the global health scene, whilst at the same time making health inequalities a priority topic, according to Harmer and Buse, ‘a paradigm shift in global health *is* underway’ [11] (p. 16). Consequently, CPGs from the BRICS countries might be candidate locations to look for discursive manifestations of this trend.

### 1.3. Heart Failure—An Emerging Topic on the Global Health Scene

In view of the specific research questions for this study, the focus on BRICS represents a useful spatial-political delimitation. In order to generate empirical answers, however, this focus must be even more narrow. Therefore, only CPGs from one specific medical field have been investigated. There are several reasons for focusing on (chronic systolic) heart failure.

Firstly, cardiovascular diseases (CVDs) are currently the leading cause of deaths in the world. With 17.9 million deaths in 2016, they account for approximately one third of annual global mortality [24]. Over three quarters of CVD-caused deaths take place in low- and middle-income countries (LMICs), where primary health care infrastructures are often underdeveloped and access to secondary care is not affordable for large parts of the population [25] (p. 54); [26] (p. 9). Contrary to the widespread belief that CVDs are diseases of the wealthy, in most countries, the poor are disproportionally affected, because they are frequently exposed to risk factors and have worse access to health care services [27] (p. 405); [28]. Heart failure is a syndrome and often final terminal stage of a range of CVDs. According to one meta-study [29], it accounts for around 2.2 percent of hospital admissions in LMICs. The same study noticed that the prognosis of heart failure patients admitted to hospitals in LMICs was on average poorer than for patients in high-income countries (HICs). Furthermore, with a mean age of 63 years, on average, the affected population in LMICs is considerably younger than in HICs. These trends are confirmed by a recent prospective study that compared heart failure worldwide [30]. This study likewise observes higher mortality rates in poorer regions of the world. In sum, worldwide and national inequality is a factor with considerable impact on the epidemiology of heart failure. It may thus be expected to be taken into account by CPGs developed in and for LMICs, such as the BRICS countries.

Secondly, as diverse and regionally varying aetiologies have been identified as underlying heart failure, this makes heart failure an interesting case for a comparison of CPGs in this field. There is far reaching consensus with regard to the definition of the syndrome. However, because of considerable interregional variation, guideline recommendations that address the underlying causes of heart failure may be expected to feature similar interregional variation.

Thirdly, guidelines on heart failure are amongst the most frequently cited guidelines published by national and regional cardiological associations. Moreover, CPGs on heart failure are, in general, amongst the most frequently cited documents in the field of heart failure research. A search on Web of Science with the query string ‘TI = “heart failure” OR TI = “cardiac failure”’ (date of search: 21 January 2020) returned a total of 103,004 documents. The European Society of Cardiology’s recent heart failure guideline [1] had been cited 4494 times, and the American College of Cardiology and the American Heart Association’s recent CPG on heart failure [31] had been cited 3867 times on the day of the search. Of all 103,004 documents, the European Society of Cardiology’s guideline was the fourth most cited in general and the most cited document after 1999. The American College of Cardiology/American Heart Association’s guideline was the sixth most cited in general and the third most cited document after 1999.

In sum, both organisations’ CPGs have clearly steered global discourses on heart failure in the years following their publication. They have thereby considerably contributed to making heart failure a topic on the global health scene. As this article shows, the heart failure guidelines of US and European professional associations are frequently referenced and considered benchmarks by many organisations from around the world, including cardiological associations from BRICS. These CPGs can thus be seen as a global CPG model for the diagnosis and treatment of heart failure. This article focuses upon whether and how the standards of a subfield of heart failure—chronic heart failure—in the BRICS-countries have been shaped by global scripts and models.

## 2. Theoretical Background

This section briefly outlines this article’s major theoretical tenets, premises and concepts hinted at in the Introduction. The sociological, neoinstitutional ‘world-polity’ theory assumes that a global level of social reality and social structure has emerged in the post-war epoch. Neoinstitutional scholars variously refer to this structure as ‘world society’ or ‘world-polity’. According to neoinstitutionalists, the world-polity constantly generates ideas, semantics, concepts, scripts and models that reach beyond the scope of nation states [6]. It consists of a repertoire of diverse actors, such as international governmental and non-governmental organisations, supranational organisations and transnational actors. The world-polity has contributed to producing and generating rationalised forms of knowledge and social models, which have spread worldwide. Consequently, Meyer and other neoinstitutionalists identified a global trend towards institutional ‘isomorphism’, i.e., an increasing convergence of ideas, standards and social models worldwide, as well as national emulation and imitation of global scripts and models [6,8].

Although the macro-sociological world-polity theory has, on the one hand, empirically demonstrated the global diffusion of Western ideas and models in various areas, e.g., in higher education, women’s education, environmental protection and science [32,33,34], neoinstitutional theorists have, on the other hand, admitted the limits of global diffusion by proposing the concept of ‘decoupling’. By decoupling, they mean that despite the empirically observable convergence at a formal and symbolic level, a gap between proclamation and implementation of normative targets within nation states has persistently existed [35]. Resource-poor states such as Global South countries can more easily adapt to global scripts and models at a symbolic level, rather than really implementing them, since a lack of resources often constrains local practices and aspirations.

Other empirical studies of global diffusion suggest that global scripts have been embedded in national socioeconomic and sociocultural contexts and that global ideas must be adapted to local circumstances by reformulation and reinterpretation through local scientific elites [36,37]. The concept of vernacularisation, proposed by anthropologists of law [38], offers a similar argument. Its proponents have noted that a wide range of factors, such as national gender ideologies, particular national histories, the complex relationship between state and society, social movements and religious traditions, shape different types of local adaption to global standards, for instance, in the arena of women’s rights [38].

For medicine and disease treatment, a global horizon of communication, perception and observation has also been looming through the worldwide diffusion of Western evidence-based biomedicine, which employs ‘rationalised’ research methods to study the human body [39]. Furthermore, pandemics such as HIV or SARS have increasingly been conceived as global risks stretching beyond national borders, and the diagnosis and treatment of diseases, along with health protection, have likewise become global issues, targeted by a cosmopolitan medical field. These ‘global regimes’ [40] are based on condensed and intensified communication and interaction of physicians, public health specialists, medical associations and diverse epistemic communities around the world [41,42,43].

This article demonstrates that global scripts, including models and standards in relation to (chronic) heart failure, have been developed by Western and international cardiological associations and have constructed a global communication context for this arena (see the section entitled A Global CPG Model). We focus on the BRICS countries in order to test the neoinstitutional approach towards global diffusion and global isomorphism outlined in this section. BRICS provides a compelling case, because its member countries are now widely considered autonomous actors with powerful nation states in the world society, resisting influences from hegemonic Western countries and organisations.

The BRICS cluster consists of a heterogeneous community of nation states with diverse economic conditions (low-income, middle-income and upper-middle-income economies), different cultures and religious traditions (Confucianism, Hinduism, Eastern Orthodoxy and Catholicism) as well as different medical traditions and health systems, e.g., the Bismarck model of social health insurance and the Beveridge model of health protection. With a focus on BRICS, this study thus undertakes a sociological and empirical test by examining whether and how Western and global scripts and models of CPGs on (chronic) heart failure have been transferred to this powerful and heterogeneous country cluster and whether an effect of convergence (or divergence) from the global models and standards can be identified against the backdrop of an inter-state comparison.

## 3. Materials and Methods

Our study is based on a comparative analysis of CPGs published by cardiological organisations from BRICS in 2018, when in four of the BRICS countries CPGs on heart failure were published. South Africa is the only BRICS country that has not recently published a CPG on heart failure. Since we understand the guidelines developed by the European Society of Cardiology/Heart Failure Association and the American College of Cardiology/American Heart Association/Heart Failure Society of America as a global CPG model for the diagnosis and treatment of chronic heart failure, we included these organisations’ CPGs as well. Table 1 gives an overview of all CPGs included in the comparison.

CPGs offer recommendations on a wide range of issues. Within the frame of our study, it was impossible to compare all recommendations for each of these issues. Therefore, from all the recommendations given in the relevant CPGs, we selected a small number of recommendations that would serve as dimensions for comparison. These recommendations appeared to be highly relevant for the current cardiological community for various reasons, for example, because they were novel, controversial, costly or relating to ‘ground-breaking’ trials. We further narrowed down these recommendations in accord with a set of parameters so as that reasonable comparisons could be made (see the following section: Dimensions of Comparison). Aside from a direct comparison of what recommendations are given in each CPG with regard to each individual dimension, a guiding question was how and whether CPGs explicitly consider national or regional particularities. Specifically, we looked for statements on particular problems associated with (global) health inequalities.

Since all of the authors of this article are sociologists, our interpretation of the CPGs in our dataset was guided by sociological methods. The qualitative and interpretative approach of grounded theory [50] allowed us to identify a set of salient and relevant categories of current discourses on heart failure and to make such categories dimensions of our comparison. Our analysis began with a review of current debates in heart failure research. At this stage, the goal was to become familiar with the most important tenets and problems of heart failure research in most general terms. We read guidelines, scientific articles and also information addressed to patients and the general public. Importantly, our analysis was also informed by insights gained from our interviews with cardiologists, all authors of CPGs, in the field of heart failure. An important advantage of conducting expert interviews over reading scientific articles is that the interviewee can be asked to elaborate on the particular topics of interest. Moreover, if necessary, interviews with experts in cardiology allowed us to ask the experts for ‘translations’ of professional language and concepts into layperson terms. This initial engagement with the current discourses on heart failure allowed us to define a number of relevant dimensions that could reasonably be compared across clinical practice guidelines from different countries. Individual members of our multilingual team then analysed each clinical practice guideline with a focus on selected dimensions. Results were then discussed in team sessions.

## 4. Dimensions of Comparison

We decided to include six different dimensions of comparison:

- the definition and distinction of types of heart failure as measures of left ventricular ejection fraction (LFEV);
- cut-off values for tests with natriuretic peptides (NP) to rule out chronic heart failure;
- standard (‘first-line’) drug treatment;
- the new angiotensin receptor blocker neprilysin inhibitor (ARNI);
- cardiac resynchronisation therapy (CRT);
- implantable cardioverter defibrillator (ICD)

We now briefly explain the rationale for selecting each of these dimensions. As already noted, our comparison does not feature the whole spectrum of recommendations for each of these issues, but only recommendations for selected parameters. The 2016 ESC Guidelines and the ACCF/AHA Guideline (with updates) were used as benchmarks here; we considered these CPGs a global model (see Introduction and Theoretical Background sections). For technical details on each of these dimensions, the reader may consult the rich cardiological literature covered in the relevant CPGs.

The ejection fraction of the heart’s left ventricle (LVEF) has long been used as a major criterion for the diagnosis of heart failure. Clinicians often use this measure to place an initial diagnosis on a more solid foundation. It also serves as a criterion for deciding on inclusion or exclusion of participants in many clinical trials. Traditionally, two types of heart failure have been distinguished according to LVEF. Heart failure with preserved ejection fraction (HFpEF) means that the LVEF is equal to or more than 50 percent. Heart failure with reduced ejection fraction (HFrEF) means that the LVEF is less than 40 percent. The range between HFrEF and HFpEF has been treated as a ‘grey area’. However, in order to foster more clinical research on this range, the ESC proposed a new definition of heart failure with middle range ejection fraction (HFmrEF) in its recent 2016 ESC Guidelines [1]. In our study, we have included LVEF as a dimension of comparison for two reasons. Firstly, LFEV is such an established concept in the cardiological community that it would have been surprising not to find it adopted in guidelines from BRICS. Secondly, because HFmrEF was an innovative definition in 2016, it was interesting to see whether it had already been picked up in recent guidelines from BRICS.

For our second dimension of comparison, we have chosen recommendations given with regard to the usage of natriuretic peptides (NPs), whose introduction into clinical practice was ‘the most important advance in the diagnosis of chronic heart failure in the last decade’ [51] (p. 1778). Since patients with chronic heart failure typically have increased levels of certain kinds of NPs, NP tests have been made available for diagnosing chronic heart failure. These tests have high negative predictive value for certain threshold values. That is, a diagnosis of chronic heart failure can be ruled out with high certainty, when the NP level is below a specific cut-off value. The ESC determined these cut-off values at 125 pg/mL for NT-proBNP, and 35 pg/mL for BNP [1]. Although the ACC/AHA/HFSA guidelines do not differ from the ESC’s definition here [52] (p. 2759), there are a number of different methods of measurements available and the definition of clear cut-off values is still a topic of debate [51]. The definition of cut-off values for NP tests thus represents a dimension of comparison in which differences across guidelines could be expected.

Our third dimension of comparison concerns recommendations for what are often referred to as ‘first-line drugs’, i.e., standard pharmacological treatment for nearly all chronic heart failure patients. In both the 2016 ESC Guidelines and the ACCF/AHA Guideline (with updates), this family of drugs consists of a combination of angiotensin-converting enzyme inhibitors (ACE-I), beta-blockers and mineralocorticoid/aldosterone receptor antagonists (MRA). In addition, diuretics are usually prescribed to relive symptoms such as congestion. In both of these guidelines, standard treatment routines follow a definite step-by-step algorithm. As van der Meer et al. observed in regard to differences between these guidelines’ recommendations for pharmacological treatment, ‘Both guidelines are highly concordant for treating HFrEF’ [52] (p. 2751). Because of the high degree of convergence between the guidelines, variations in guidelines from BRICS would prove surprising and all the more compelling. 

The fourth dimension of comparison concerns recommendations offered for treatment with ARNIs. The emergence of ARNIs on the global pharmacological landscape dates back to the outcomes of a study referred to as the Prospective Comparison of ARNI with ACEI to Determine Impact on Global Mortality and Morbidity in Heart Failure Trial (PARADIGM-HF) [53]. This randomised controlled trial was celebrated by the heart failure community as paradigm changing. The study’s results have allowed regulatory agencies in many countries to approve a new drug for chronic heart failure treatment. Treatment recommendations had to be included in recent guidelines; thus, ACC/AHA/HFSA put out updates in 2016 and 2017. These updates recommend ARNIs for patients with chronic symptomatic HFrEF that tolerate an ACE-I or ARB [44,45]. Since the ESC writing committee convened earlier than the US committee, that is, before first clinical experiences with the new drug had been made outside of PARADIGM-HF, the 2016 ESC Guidelines recommend replacement for ACE-I only if a patient remains symptomatic despite optimal medical treatment with ACE-I, beta-blocker, and MRA [1]. These recommendations are more cautious than those given by the US organisations, as several guideline authors noted in our series of expert interviews [54,55]. Because of these slight differences, ARNI was an interesting case for our comparison. Did guidelines from BRICS follow the more cautious but meanwhile outdated recommendation of the ESC? Or did they follow the ACC/AHA/HFSA? Or perhaps neither of these?

The last two dimensions for comparison are specifically relevant to our concern with health inequalities, as the recommended device therapies tend to be costly. With our fifth dimension of comparison, CRT, we concentrated on a recommendation published in both the ESC and the ACC/AHA guidelines, regarded as a Class I recommendation with the highest level of evidence [56]. Both the ACCF/AHA Guideline and the 2016 ESC Guidelines recommend CRT for symptomatic patients with left bundle branch block, when the patient’s QRS (Q, R, and S waves) is equal to or more than 150 ms, LVEF is equal to or less than 35 percent and symptoms have not improved despite optimal medical treatment [1]. There are some interesting conditions specified that serve as contraindications. ESC and ACC/AHA use different cut-off values here. In the 2016 ESC Guidelines, a QRS duration less than 130 ms is said to be an indicator against CRT. The ACCF/AHA Guideline, however, defines the respective cut-off value to be 120 ms [31]; [52] (p. 2763). Because of this variation, it is interesting to see how guidelines from BRICS define the respective cut-off value (if they do so at all).

We have selected the recommendation for ICD implantation as the sixth dimension of comparison. ICDs are implanted in order to reduce patients’ risk of mortality, especially the risk of sudden cardiac death. Guidelines from the ESC/HFA and ACC/AHA/HFSA are widely concordant in this domain [52] (p. 2762). They recommend ICD for primary prevention in patients with symptomatic chronic heart failure, when the LVEF is less or equal to 35 percent and symptoms have not improved despite at least three months of optimal medical treatment. An ICD should not be implanted when the patient has suffered a myocardial infarct in the past 40 days or if the patient is not expected to live more than one full year [1,31].

## 5. A Global CPG Model

CPGs are a scientific genre anchored in the concept of evidence-based medicine (EBM). They are used by health organisations around the world. The genre’s origins, however, like the origins of the EBM movement itself, are in Western countries, specifically in the United States, Scandinavia and Europe [57,58]. Our literature search revealed that the first CPGs on heart failure were developed in Europe and the United States in the early 1990s. As the following brief review about the history of CPGs on heart failure indicates, an (informal) coalition of US and European professional associations, which might be referred to as the ‘transatlantic five’ of heart failure research, still dominates the global generation of knowledge and discourses on heart failure. CPGs developed by these cardiological organisations are one of the tools that help reproduce their epistemic dominance.

In the first half of the 1990s, the ESC and the ACC, together with the AHA, were amongst the first cardiological organisations that developed guidelines on CVDs. Before these organisations initiated their series of CPGs on heart failure, the US Agency for Health Care Policy and Research (AHCPR) had already published a CPG on the topic in 1994 [59]. However, 1995 stands out as the real watershed for heart failure guidelines. In this year, the ESC published its first CPG specifically dedicated to heart failure [60], after the Working Group on Heart Failure had recently been founded (which would later become the Heart Failure Association). This document covered only recommendations for the diagnosis of heart failure. A separate CPG on treatment came out only in 1997. In 1995, however, the ACC’s collaboration with a task force from the AHA resulted in another CPG on heart failure, which was the first CPG published by these organisations on the topic [61]. Moreover, 1995 also saw the incorporation of the HFSA, an American pendant to the HFA, but in contrast to the latter, it was independent from larger cardiological associations. From 1995 on, all of these organisations, except for the AHCPR, independently pursued their own lines of regularly updated CPGs on heart failure.

In 2016, their work had become substantially aligned, however. The HFSA had become co-editor of the updates to the ACCF/AHA Guideline. There is thus currently only one CPG on heart failure in the United States. Moreover, joint editorials by ESC, HFA, ACC, AHA and HFSA [62], which accompanied the 2016 ESC Guidelines and the US update from 2016, underlined that there is a transatlantic aspect to this organisational alignment. For one, cardiological congresses, as well as research and writing collaborations between individual members from the United States and Europe, serve the exchange of ideas on a more informal level. However, organisational collaboration proceeds on a formal level as well. For example, writing committees on both sides of the Atlantic stay in touch with each other concerning guideline development through member exchange. The recent ESC writing committee had an eminent US cardiologist and ACC/AHA Fellow, Mariell Jessup, on board. Conversely, Gerasimos Filippatos, a European heart failure expert and ESC Fellow, was a member of the ACC/AHA/HFSA’s writing committee for the updates of 2016 and 2017. Moreover, after the drafts for publication in 2016 had initially been developed concurrently by the US and the European committees, they were then shared before publication [62] (p. 1474).

Organisational alignments thus have repercussions on guideline convergence, as has been noted by the cardiological community itself [52]. The differences that remain in regard to particular guideline recommendations, e.g., for the ARNIs or CRT, are mainly attributed to time lags that often exist ‘between production of evidence and its incorporation into guidelines’ [56] (p. 314).

Cardiologists and guideline authors themselves frequently account for guideline convergence by referring to a quasi-positivistic theory of knowledge. In a series of interviews with cardiologists that had been involved in guideline development, we asked if they had witnessed any alignments between US and European guidelines. Their answers were affirmative across the board. When pressed for an explanation, all of the interviewees provided accounts that were more or less similar to the following (quoted verbatim from an interview with one of the authors of the 2016 ESC Guidelines):

Well, let’s assume that science is about finding the truth. And that there is a truth for a particular question in the end. If the whole system of science works, things will necessarily move from the uncertain and questionable into the direction of that truth … And I think that this is the reason why guidelines have more and more converged over the last decades. [63]

An assumption underpinning this idea is that many clinical trials are now ‘evidence-based’. They thus enable researchers to work out the contours of a constant and stable truth in regard to particular questions. Guideline convergence is thus a necessary consequence of inexorable scientific progress. Other cardiologists provided similar quasi-positivistic accounts. At the same time, however, many of our interviewees also noted the fact that the guidelines developed for US and European populations might not be applicable to other regions of the world. For instance, although the above-quoted cardiologist noted that big clinical studies are mostly international now and study cohorts thereby represent mixed backgrounds, he also noted that guidelines developed by the ESC/HFA or the ACC/AHA/HFSA might not be applicable to certain populations in Asia or Africa.

Notwithstanding such doubts, over the last three decades, the transatlantic five of ESC/HFA and ACC/AHA/HFSA have been able to establish themselves as ‘policy leaders’ [8] (p. 456) in the global field of heart failure. Their CPGs function as global scripts and a global CPG model that shapes the discourses and knowledge of the global cardiological community.

## 6. Uptake of the Global Model in BRICS

In this section, we examine in detail how cardiological associations from BRICS have positioned themselves in regard to the global CPG model on heart failure with a special focus on chronic heart failure. All of the CPGs included in our comparison frequently refer to the ACCF/AHA Guideline and/or the 2016 ESC Guidelines, sometimes in terms of a comparison with their own recommendations, often also in terms of a benchmark. At the same time, the CPGs from BRICS also note specific demands for national adaptation.

Table 2 highlights the results of the comparison. Since the South African Heart Association has not published any recent CPG on heart failure, but instead endorses the ESC Guidelines, South Africa is not included in this table.

A first glance reveals that recommendations given in the CPGs from BRICS are overwhelmingly concordant with those given in what we have identified as the global CPG model. Sometimes CPGs from BRICS refer to the ACCF/AHA Guideline recommendations as a benchmark; at other times, they refer to the 2016 ESC Guidelines. Oftentimes, they reference both groups. Why do some recommendations given in CPGs from BRICS refer to the European guidelines rather than to the US guidelines, whilst the opposite is true in case of some other recommendations? Certainly, there is a number of explanatory factors, but in our view the most important one is likely time lags between the publications of the guidelines that constitute the global CPG model, as is also suggested by some of the cardiologists interviewed by us (see the section entitled A Global CPG Model). For instance, the recent ESC Guidelines came out very soon after the ground-breaking results of PARADIGM-HF had been published. PARADIGM-HF made a strong case for ARNIs to substitute ACE-inhibitors. However, ARNIs were not yet on the market and PARADIGM-HF had also identified certain risks and uncertainties associated with the drug. The ESC Guidelines were thus a little bit more cautious in recommending substitution of ACE-I with ARNIs than the US guideline updates, which came out later. When national cardiac organisations from BRICS brought out their guidelines, in 2018, first experiences with ARNIs had been made outside the confines of the PARADIGM-HF trial. Hence, risks and benefits could be evaluated on a firmer base of evidence and, accordingly, recommendations in guidelines from BRICS were referencing the more recent US updates rather than the already (slightly) outdated ESC Guidelines. Interestingly, in 2019, the HFA published a clinical practice update in which recommendations concerning treatment with ARNIs were more aligned with the US updates [65].

Although we observed a general trend of convergence of guideline recommendations, there are some variations and deviations from this observation. We discuss these in the following subsections, where we also further contextualise each of the relevant CPGs.

### 6.1. Brazil

The Brazilian Cardiological Society (BSC) was founded in 1943 as the fifth professional cardiological association in America. According to the organisation’s ‘official history’ [66], initially, many Brazilian physicians were heavily influenced by specialist literature from Europe. The organisation’s archives also bear witness of some active collaborations in this period. After World War II had delimited Brazilian cardiologists’ access to Europe, the influence of the US cardiological community on Brazilian cardiology began to grow.

The BSC has a relatively long tradition of guideline development, with a first consensus paper on heart failure being published in 1992 [67]. The Diretriz Brasileira, included in our comparison, was published in 2018. As can be seen in Table 2, the recommendations in this CPG largely converge with those of the global CPG model.

With regard to the first comparative dimension, that is, the differentiation of heart failure by levels of LVEF, the ESC’s definition was used. The definition of heart failure is thus in accord with the ESC’s definition of the new class of HFmrEF. Concerning the specification of cut-off values for the diagnostic exclusion of chronic heart failure through NP testing, the Brazilian guideline is indecisive between the definition from the ESC and that given in a Canadian CPG [65]. The Diretriz Brasileira argues that a BNP cut-off value of 50 pg/mL would imply greater specificity and thereby reduce the number of false-positive diagnoses. In contrast, the ESC’s definition of a cut-off value for BNP above 35 pg/mL would allow for greater sensitivity, thereby reducing the number of false-negative diagnoses, and make necessary additional examination of patients actually not affected by chronic heart failure [46] (p. 445). With regard to ARNI, the Diretriz Brasileira recommends replacing ARNI for ACE-I for any patient with HFrEF or HFpEP, if the patient has been stabilised already. The decision for this recommendation is based on the PARADIGM-HF study, and it is rather oriented towards the recommendations from the updated ACCF/AHA Guideline [46] (p. 457). In terms of first-line drug treatment, the Diretriz Brasileira largely makes recommendations that are similar to those in the 2016 ESC Guidelines. The same applies to CRT and ICD, in which the 2016 ESC Guidelines and the ACCF/AHA Guideline do not differ, at least in regard to the parameters selected for our comparison.

Interestingly, the Diretriz Brasileira includes a whole chapter on the specific aetiology of heart failure in Brazil. Special attention is directed to the Chagas disease, which is, according to the WHO, a ‘disease of the poor’ [25] (p. 66). In Brazil, the mortality of patients with chagasic heart failure can reach 20 percent per year. These patients’ prognosis is worse than that of patients with secondary heart failure caused by ischemic and non-ischemic diseases. However, the Diretriz Brasileira recommends pharmacological treatment strategies that are similar to those for non-chagasic heart failure patients [46] (p. 472).

Some passages in the Diretriz Brasileira deal with considerations of medical costs, as Brazil’s health system suffers from low investment, inadequate access to health care facilities, etc. [46] (p. 444). Nevertheless, all Class I recommendations with Level A evidence are considered cost-effective, when considering the disposal thresholds from developed countries. Besides the ‘rational’ use of health resources, the guideline recommends providing the greatest benefits to the largest number of patients [46] (p. 482).

Overall, the Diretriz Brasileira widely converges with the global model of heart failure. Our qualitative analysis of the document confirmed this finding; it revealed that the Diretriz Brasileira frequently makes references to the ACCF/AHA Guideline and the 2016 ESC Guidelines in connection with recommendations other than those included in our comparison. However, the Diretriz Brasileira does not adopt the global CPG model outright. Rather, the recommendations and definitions in the Diretriz Brasileira partly deviate from this model and, perhaps more importantly, they assign a substantial amount of space to some particularities of heart failure in Brazil. Thus, even though the global model’s relevance for the development of the Diretriz Brasileira is certainly without doubt, it has been to some extent adapted to the specific conditions of Brazil.

### 6.2. Russia

Because the Russian Society of Cardiology (RSC), established in 1963, has been a member of the ESC since 1993, CPGs developed by the RSC are somewhat a special case within the framework of our comparison. The RSC officially endorses the 2016 ESC Guidelines. The translation of the latter into Russian was published in Rossijskij kardiologičeskij žurnal, the RSC’s official publication organ [68]. That is, the RSC adopted the ESC guideline outright and adaptation in this case is reduced to the process of translation. There is a second CPG on heart failure, however, which was also developed by the RSC, but in collaboration with the Russian Society of Heart Failure Specialists (SHFS) and the Russian Scientific Medical Society of Internal Medicine (RSSIM) [47]. The Kliničeskie rekomendacii are not a mere translation of the 2016 ESC Guidelines, even though the document explicitly states that it is based upon CPGs published by the ESC in 2005, 2008, 2012 and 2016. Additionally, it refers to CPGs published by ACC/AHA in 2005, 2009 and 2013, and the corresponding updates from 2016 and 2017 as benchmarks [47] (p. 8). However, the document qualifies this statement of endorsement, as it features ‘a number of refinements, additions and changes that take into account both national particularities and a somewhat different interpretation of some of the debatable propositions of some major multicentre studies’ [47] (p. 10).

Since the RSC’s translation of the ESC 2016 Guidelines is identical with the latter, our analysis of selected recommendations concentrates on the Kliničeskie rekomendacii. As shown in Table 2, in this CPG, the definition for forms of heart failure by means of LVEF is identical with the definition given in the 2016 ESC Guidelines. The ESC’s new definition of HFmrEF is also introduced in the Kliničeskie rekomendacii. Likewise, for ruling out chronic heart failure with NP tests, the Kliničeskie rekomendacii specified cut-off values in line with the values specified in the 2016 ESC Guidelines. As in the 2016 ESC Guidelines, first-line treatment consisted of ACE-I, beta-blockers and mineralocorticoid receptor antagonists. Levels of evidence and classes of recommendation for each of these particular recommendations were largely identical with those given in the ESC Guidelines. Only in regard to ARNI, the Kliničeskie rekomendacii recommend replacement for ACE-I in any patient with HFrEF or HFpEP, if the patient was stabilised already. This recommendation is more in line with the recommendation given by ACC/AHA/HFSA. This is probably due to the fact that the Kliničeskie rekomendacii came out two years after the ESC guidelines and the writing committee thus had additional clinical experience with ARNI at their disposal.

Notwithstanding their brief note about national specificities, the Kliničeskie rekomendacii do not really consider national-level particularities, e.g., in terms of Russia’s special demographic and epidemiological conditions, specific aetiologies of heart failure, (lack of) accessibility and affordability to health care services and so on. In sum, in case of the Kliničeskie rekomendacii there are strong indications for extensive adoption of the global model and rather less adaptation.

### 6.3. India

In the Indian case, there are a number of recent documents devoted to providing, in one way or another, guidance for the diagnosis and treatment of chronic heart failure. We singled out the CSI Position Statement as the most comprehensive of these documents. It was written by a group of experts based at Indian institutions and published in the Indian Heart Journal, the official journal of the Cardiological Society of India (CSI). Even if it modestly avoids the word ‘guideline’ in the title, this document has all of the usual components of a regular CPG: a large group of experts in cardiology (the paper has more than 70 authors) contributed to it, it is based on a formalised writing process with writing committee members reaching consensus on individual recommendations through discussion and vote, it states its purpose to provide guidance to clinicians for the whole country etc. The document’s structure also very much resembles CPGs issued by the dominant cardiological organisations: a preamble, discussion of the epidemiology and aetiology, its (economic) burden on health systems, a section on the diagnosis of chronic heart failure followed by a section on its treatment/management etc. Because of that, it could rightfully be considered a CPG.

The CSI Position Statement takes a somewhat critical position towards ‘Western guidelines’ on heart failure diagnosis and treatment. It states the following:

A number of guidelines, mostly Western, are available for management of patients with this condition but their uniform implementation may be difficult across all countries because of differences in infrastructure and local practices. Therefore, there is a need for region specific and country-specific guidelines. [48] (p. 5)

For one, the CSI Position Statement notes the specific demographic and epidemiological situation of India, where chronic heart failure patients are on average much younger than in Western countries. Secondly, the typical aetiology of chronic heart failure in India differs, since rheumatic heart disease is a common cause. Thirdly, the CSI Position Statement acknowledges the striking inequalities in terms of access to health care services in India. Certain recommendations for cost-intensive treatment made in the Western guidelines simply would not be affordable for major parts of the Indian population [48] (p. 6). Fourthly, the CSI Position Statement notes that Western guidelines are mostly based on clinical studies with ‘Caucasian’ populations. In contrast, data for the typical Indian patient are lacking. Guideline recommendations thus cannot draw from the same evidential base from which Western guidelines draw [48] (p. 6).

As Table 2 reveals, for the first three dimensions of comparison, the recommendations from the CSI Position Statement are largely aligned with the ESC/HFA and ACC/AHA/HFSA guidelines. The recommendation in regard to the prescription of ARNIs is somewhat a mixture of the respective recommendations from the ESC and ACC/AHA/HFSA: ARNIs are recommended when symptoms do not improve despite optimal medical treatment with the first-line drugs (ACE-I, beta-blocker, MRA); they are also recommended as replacement for ACE-I on an individual basis for patients who already tolerate treatment with ACE-I. However, the more interesting divergences from the global CPG model concern recommendations for CRT and ICD. The CSI Position Statement discusses at length the specific challenges CRT and ICD pose for settings with limited resources. It states the need to ‘develop … own guidelines for device based therapy in HF in a resource limited setting taking into account ethnic, socio-economic and geological factors’ [48] (p. 29). The recommendations given by the ACC/AHA for CRT, even in patients with NYHA Class I, are seen as ‘overenthusiastic’ for the case of India. The CSI Position Statement is thus similar to both the 2016 ESC Guidelines and the ACCF/AHA Guideline, but it restricts CRT to patients with NYHA Class III and certain ambulatory patient with NYHA Class IV [48] (p. 30). In regard to ICD, the CSI Position Statement stresses the dimension of economic affordability. Although the general recommendation aligns with ESC and ACC/AHA guidelines, it is clearly stated that ‘[t]hese are recommendations for primary prevention in Western guidelines but for a country like India, these should be individualised and financial resources of the patient and the government reimbursement also needs to be factored in’ [48] (p. 30).

In sum, amongst all the CPGs included in our comparison, the CSI Position Statement is the one with the highest degree of adaptation. Frequent references in the guideline to the aetiological, demographic, and socio-economic particularities of India point in this direction as well. The CSI Position Statement is also the only CPG that explicitly and repeatedly mentions health inequalities and lack of accessibility to health care services and technologies as issues that make guideline transfer difficult. Therefore, it is all the more compelling that the global CPG model has had nonetheless a seemingly huge impact on the formulation of the CSI Position Statement; despite the observed deviations and variations, most of the investigated recommendations remain in line with the global model.

### 6.4. China

In China, a few influential institutes and agencies in the cardiological field have been actively engaged in formulating standards for the diagnosis and treatment of heart failure. One of the most relevant actors is the Chinese Society of Cardiology (CSC), founded in 1978. It is a central agency in the area of heart diseases which has coordinated with some other organisations and institutions on agenda and standard setting for this special field.

Following the guidelines proposed by ESC/HFA and ACC/AHA/HFSA, the CSC and its subordinate Chinese Journal of Cardiology (CJC) initiated the Guideline for Diagnosis and Treatment of Heart Failure in China 2014 [69], which had two predecessors, a CPG on chronic heart failure and a separate CPG on acute heart failure. In 2018, after a careful review of the new heart failure guidelines from the transatlantic context, the CSC collaborated with a special professional committee of the Chinese Medical Doctor Association (CMDA) and the CJC, and created the updated Guideline for Diagnosis and Treatment of Heart Failure in China 2018 [49]. This new document is currently considered the latest and most authoritative document in the cardiological field concerning heart failure in China.

The physicians and professors of the CSC we interviewed underscored the fact that the Chinese cardiological/heart failure community has deliberately noted the development of the update and the new trends promoted by their European and American colleagues, integrating state-of-the-art knowledge from the ESC and the ACC/AHA into the 2018 version of the guidelines. Although some interviewees mentioned the Canadian guideline and guidelines from other Asian countries at the margin, nearly all interviewees highlighted the significance of the updated versions of heart failure guidelines proposed by the ESC and the ACC/AHA, which constitute the primary reference frameworks for the Chinese cardiological community [70,71].

Table 2 reveals that along our selected dimensions, the Chinese cardiological community is oriented mostly towards the guidelines of the ESC/HFA and the ACC/AHA/HFSA. In the definition of heart failure, China adopts the new criteria proposed by the ESC, namely integration of heart failure with middle range ejection fraction (HFmrEF) into the definition of heart failure. Pertinent to the cut-off values for tests with natriuretic peptides to rule out chronic heart failure, China also follows the standard of the ESC (Table 2). In the dimension of ARNI, the Chinese cardiologists connect to the latest development from the North American context and recommend the replacement of AEC-I/ARB by ARNI for patients who already tolerate treatment with ACE-I and ARB. In other cases, the Chinese guideline is connected to both the ESC and the ACC, such as the application of medical devices like CRT and ICD (Table 2).

Apart from the dimensions included in our comparison, the Chinese guideline also devotes some space to Chinese medical developments and ingredients, such as the use of evidence-based Chinese herbal medicine, including Qili, an herbal medicine for the treatment of chronic heart failure. The Chinese guideline also discusses some special conditions underlying heart failure in certain patients, such as China’s high-altitude plateaus and cohort-based chronic heart failure with a focus on the country’s elderly and most elderly population [49].

In sum, very much like the Brazilian CPG, the Chinese HF Guideline 2018 does not completely copy the global CPG model. Its recommendations partly deviate from the global model and, like the Brazilian CPG, it devotes substantial space to a discussion of particularities of chronic heart failure in China. Nevertheless, the important role of the ACCF/AHA Guideline, and perhaps even more the role of the 2016 ESC Guidelines as benchmarks and models is evident in the Chinese heart failure Guideline 2018.

### 6.5. South Africa

South Africa is one of the few countries in which medical organisations published a CPG on heart failure already in the last millennium [72]. The South African Heart Association (SA Heart) was created in 1999 from two cardiological predecessor organisations. It has been an associate member of the ESC since 2005. In 2005, it also created the Heart Failure Society of South Africa (HeFSSA) as a special interest group. Despite early interest in CPGs on heart failure in South Africa, in recent years, these cardiological organisations have not been active in developing their own CPGs. Instead, ‘SA Heart^®^ subscribes to the guidelines issued by the European Society of Cardiology [but] publishes position papers or commentary on guidelines for local circumstances where appropriate’ [73].

The last of these position papers was published by the HeFSSA in 2013, referring to the ESC 2012 guideline. Similar to the Indian guideline document discussed above, this position paper recommended natriuretic peptides tests for chronic heart failure diagnosis in case of limited availability of echocardiography [74] (p. 662), thereby at least recognising the dimension of health inequality. Possibly surprising however, this is the only place in the position paper where health inequality is a topic. In a country which has one of the highest levels of inequality worldwide [75], with the distribution of health services clearly skewed towards the richer parts of the population [76], health inequalities might be expected to occupy a more prominent place in the position paper.

Similarly, other South African particularities, such as the specific epidemiology and aetiology of heart failure in this country, are equally absent in the position paper. This is despite studies and voices from the cardiological community that have addressed the need for South African-specific guidelines. For instance, a big clinical study in the township of Soweto had earlier shown several patterns uncommon for high-income countries but deemed highly relevant for South Africa’s transforming urban areas. Amongst other things, the study had observed that its cohort was largely comprised of ‘young black African women’ and that study participants suffered from a ‘broad diversity of heart disease attributable to a combination of infectious and non-communicable diseases’ [77] (p. 2365). As the Soweto population was deemed to be situated in a so-called ‘epidemiological transition’, the paper explicitly noted ‘the challenge of adapting and prioritising therapeutic strategies according to the changing etiology of HF’ [77] (p. 2366). Additionally, in accordance with its observation that amongst the study cohort there was a relatively high prevalence of a form of heart failure rarely met in high-income countries (‘right heart failure’) but typical for Soweto, it suggested the development of specific treatment strategies [77] (p. 2366). Except for the abovementioned position paper from 2013 (which does not extensively engage with such national specificities), there are no other CPGs or position papers published by South African cardiological organisations addressing these questions.

Despite a number of substantial reasons that would actually make the development of specific and tailor-made guidelines for South Africa likely, the SA Heart’s policy of endorsing ESC guidelines is thus the clearest case for a wholesale adoption of the global model within BRICS.

## 7. Discussion and Conclusions

To date, no studies published in the social sciences have examined the cross-country convergence of clinical practice guidelines. A main goal of our qualitative study was thus to show whether and how CPGs developed in BRICS countries for the diagnosis and treatment of chronic heart failure follow global scripts, models and standards, and thereby converge with these and with one another. The results of our theory-guided empirical investigation seem to support some but not all of the assumptions made by proponents of neoinstitutional world-polity theory.

Although our comparison of specific guideline recommendations was far from comprehensive, we were able to demonstrate high degrees of convergence for a number of selected guideline recommendations. By and large, the guidelines or quasi-guidelines (as in the Indian case) from the countries included in our comparison appeared to be deeply influenced and shaped by a global CPG model.

In particular, high degrees of convergence were found in recommendations in regard to the ESC’s new definition of HFmrEF, along with recommendations for first-line drug treatment, a new pharmaceutical product (ARNI), and recommendations regarding the definition of cut-off values for tests with natriuretic peptides in order to rule out chronic heart failure. Even the timing of the adoption of the new guidelines bears witness to a diffusion effect, as most of the BRICS countries followed the 2016 ESC Guidelines and the 2017 updated ACCF/AHA Guideline, initiating their own new guidelines in 2018. The developments of the technologically and economically advanced Global North have provided benchmarks and models for other countries to follow.

The findings of our empirical study also suggest that despite the socioeconomic and cultural heterogeneity of the BRICS country cluster, the essential core of the heart failure guidelines or quasi-guidelines from this cluster largely converges amongst the individual countries. This is hardly surprising, however, since all of the BRICS countries have more or less adopted a global CPG model. This is a model consisting of guidelines and incorporated standards proposed by just a handful of cardiological associations. The informal coalition of these ‘transatlantic five’ regularly publishes guidelines that constitute global reference frameworks and benchmarks for the setting of standards in the field of (chronic) heart failure. They have a massive impact on the development of new CPGs in countries and regions all over the world, including such large and influential transition countries such as BRICS. The transatlantic five have in fact assumed the role of a global actor and cosmopolitan discourse-maker that constantly generates state-of-the-art medical knowledge, based on the newest clinical research and trials. They continuously create new data sources for cardiological communities in all parts of the world and for the global cardiological community.

What about the role of other actors operating on a global scale, such as multi-national manufacturers of pharmaceuticals or medical devices? As we have also observed, the globalisation of the pharmaceutical industry has exerted partly massive influence on discourses, scripts and models related to development of clinical practice guidelines [78]. Without doubt, the pharmaceutical and the medtech industry are important factors that need to be taken into account if one’s goal is to develop a comprehensive analysis of the current conditions of evidence-based medical knowledge. These industries’ influence on decisions being made at numerous levels—beginning at the early stages of trial design, ending at healthcare professionals’ treatment decisions and direct-to-consumer advertising—has been thoroughly investigated already and studies have provided important insights as to the complex interactions between biomedicine and the economy [2,79,80]. Industrial influence certainly also reaches into guideline development, as, for instance, authors of CPGs frequently entertain relations with pharmaceutical companies [81,82]. Interestingly, this vexed relationship has given rise to a practice in guidelines to provide detailed statements concerning each author’s declaration of conflicts of interests. Perhaps originally pioneered by organisations in the West, such ‘disclosure forms’, in turn, seem to have become a specific (stylistic) item of CPGs, which is being emulated and ‘copied’ by other organisations around the world (amongst the BRICS, the Brazilian CPG provides a detailed declaration of conflicts of interests). Whether such discursive safety devices can effectively prevent industrial influence on guideline development, still remains underexplored. This is an intriguing question for further research. In this article, however, we have been concerned with the constitution and spread of a global CPG model. We identified the ‘transatlantic five’ as a number of organisational actors in the field of heart failure that have the power to steer discourses and substantially define the tenets of current knowledge about heart failure. Even if big multi-national companies operate important levers on the backstage, so to speak, it is primarily the aforementioned cardiological organisations to which authority and expertise with regard to the knowledge in question is attributed. Hence, we understand these organisations’ epistemic authority to be an important driver of isomorphism and convergence concerning CPGs worldwide and amongst the cardiac organisations from BRICS in particular.

The findings of our cross-country comparison of the five powerful and influential transitional economies of BRICS thus widely support the explanatory power of the sociological, neoinstitutional world-polity theory. Furthermore, they have helped identify a sort of ‘medical isomorphism’ amongst the BRICS countries, i.e., basically converging developments of heart failure guidelines within the selected country cluster. Consistent with neoinstitutional assumptions, worldwide isomorphic structures of clinical practice guidelines can be understood as resulting from the transatlantic five’s global epistemic hegemony in the field of heart failure.

Notwithstanding this overriding trend, we have identified some differences and unequal developments in BRICS as well. Measured by the degree of convergence with the global, namely, in this case transatlantic standards, the Russian cardiological community is the closest to the global script or model. Amongst the cardiological associations from BRICS, the Russian Cardiological Society is the only full national member society of the ESC. This in part explains why the Russian guideline diverges the least from the global CPG model.

With China’s full integration into the world economy and international community, the Chinese cardiological community has also exhibited an open attitude towards the global scripts through rationalistic learning and absorbing advanced knowledge from its European and American colleagues. At the same time, the Chinese guideline has simultaneously incorporated some specifically Chinese elements, such as a pharmaceutical product based on traditional herbal medicine. This guideline also includes sections on specific groups of patients, such as patients that suffer from chronic heart failure in the vast, high-altitude plateau areas of China.

Quite similar to the Chinese health organisations, the Brazilian Cardiological Society widely adopted recommendations from the global CPG model. In addition, very much like the Chinese guideline, the Brazilian guideline allocated space to questions in regard to the treatment of chronic heart failure that are specific to Brazil, even though the significance of these considerations for clinical practice certainly remains to be shown. Moreover, the Brazilian guideline to a certain extent also discussed questions of cost-efficient treatment and affordability.

The Indian CSI Position Statement comes closest to what can be described as an instance of (discursive) decoupling: although the position statement has blended the knowledge and concepts from the ESC and the ACC/AHA, Indian chronic heart failure experts most clearly acknowledge the fact that the recommendations provided in Western guidelines are in many ways unrealistic for the specific national context. This awareness may partly be due to the fact that India is the country with the lowest per-capita-income amongst the BRICS and also the country with the lowest percentage of spending for public health care [83].

Finally, SA Heart is an associated member of the ESC and follows an official policy of endorsing ESC guidelines. South African cardiologists have argued for the need to develop guidelines that take into account the particularities of heart failure in this country. However, apart from some older position papers, there are no recent documents that deal with such particularities. Along with the case of the Russian guideline discussed in this article, the South African case is thus the clearest instance of wholesale adoption of the global CPG model.

As stated in the Introduction, we expected worldwide and intra-country health inequalities [84], along with topics such as universal health coverage [85], affordability of medicines and technologies, to be featured in guidelines from the BRICS countries. Harmer and Buse’s suggestion [11] that BRICS is driving a paradigm change in global health, made these expectations seem reasonable. Our finding that only the CPG from India, and to less extent also the Brazilian CPG, allocated extensive space to such considerations was thus surprising. Are the other national heart failure associations and communities ‘decoupled’ from these trends? The presumed paradigm change may not yet have reached such specialist communities. On the other hand, in view of the particular economic conditions in India, it is less surprising that amongst the guidelines investigated from BRICS countries, it was the CSI Position Statement that raised special attention to health inequalities.

The global medical scripts and models of clinical practice guidelines for the diagnosis and treatment of chronic heart failure that we identified in this article have thus spread from the North to the South. Their core elements have been adopted by all the cardiological associations from BRICS included in our study. However, these organisations also variously adapted, localised or vernacularised the global CPG model. 

## Figures and Tables

**Table 1:** Clinical practice guidelines included in the comparison.

Short form Used in this Article	Publishing Organisation(s)
2016 ESC Guidelines [1]	ESC ^1^, HFA ^2^
ACCF/AHA Guideline (with updates) [31,44,45]	ACC ^3^, AHA ^4^, HFSA ^5^
Diretriz Brasileira [46]	BSC ^6^
Kliničeskie rekomendacii [47]	SHFS ^7^, RSC ^8^, RSSIM ^9^
CSI Position Statement [48]	CSI ^10^
Chinese HF Guideline 2018 [49]	CSC ^11^, HFPC ^12^ of the CMDA ^13^, CJC ^14^

^1^ European Society of Cardiology; ^2^ Heart Failure Association; ^3^ American College of Cardiology; ^4^ American Heart Association; ^5^ Heart Failure Society of America; ^6^ Brazilian Society of Cardiology; ^7^ Society of Heart Failure Specialists; ^8^ Russian Society of Cardiology; ^9^ Russian Scientific Medical Society of Internal Medicine; ^10^ Cardiological Society of India; ^11^ Chinese Society of Cardiology; ^12^ Heart Failure Professional Community; ^13^ Chinese Medical Doctor Association; ^14^ Chinese Journal of Cardiology.

**Table 2:** Selected recommendations from recent CPGs on (chronic) heart failure (HF) ^1^.

Dimension of Comparison	Brazil [46]	Russia [47]	India [48]	China [49]
*Types of HF defined through LVEF*	like ESC	like ESC	like ESC	like ESC
*Rule-out HF with NP-test*	partly like ESC, partly like CCS	like ESC	like ESC	like ESC
*First-line drug treatment*	like ESC and ACC	like ESC and ACC	like ESC and ACC	like ESC and ACC
*ARNI*	like ACC	like ACC	partly like ESC, partly like ACC	like ACC
*CRT*	like ESC and ACC	like ESC and ACC	like ESC and ACC, but only NYHA III	like ESC and ACC
*ICD*	like ESC and ACC	like ESC and ACC	like ESC and ACC, but with limitations	like ESC and ACC

^1^ ESC denotes the 2016 ESC Guidelines, ACC denotes the ACCF/AHA Guideline (with updates). CCS denotes the CPG published by the Canadian Cardiovascular Society in 2017 [64].

## References

[1] Ponikowski P., Voors A.A., Anker S.D., Bueno H., Cleland J.G.F., Coats A.J.S., Falk V., Gonzalez-Juanatey J.R., Harjola V.P., Jankowska E.A. (2016). 2016 ESC Guidelines for the diagnosis and treatment of acute and chronic heart failure: The Task Force for the diagnosis and treatment of acute and chronic heart failure of the European Society of Cardiology (ESC)Developed with the special contribution of the Heart Failure Association (HFA) of the ESC. Eur. Heart J..

[2] Lock M.M., Nguyen V.-K. (2018). An Anthropology of Biomedicine.

[3] Mishra S., Chaturvedi V. (2015). Are western guidelines good enough for Indians? My name is Borat. Indian Heart J..

[4] Dizon J.M., Machingaidze S., Grimmer K. (2016). To adopt, to adapt, or to contextualise? The big question in clinical practice guideline development. BMC Res. Notes.

[5] McCaul M., Clarke M., Bruijns S.R., Hodkinson P.W., de Waal B., Pigoga J., Wallis L.A., Young T. (2018). Global emergency care clinical practice guidelines: A landscape analysis. Afr. J. Emerg. Med..

[6] Meyer J.W., Boli J., Thomas G.M., Ramirez F.O. (1997). World society and the nation-state. Am. J. Sociol..

[7] Holzinger K., Knill C. (2005). Causes and conditions of cross-national policy convergence. J. Eur. Public Policy.

[8] Dobbin F., Simmons B., Garrett G. (2007). The global diffusion of public policies: Social construction, coercion, competition, or learning?. Annu. Rev. Sociol..

[9] United Nations Conference on Trade and Development (2019). Handbook of Statistics. https://unctad.org/en/PublicationsLibrary/tdstat43_en.pdf.

[10] Suchodolski S.G., Demeulemeester J.M. (2018). The BRICS Coming of Age and the New Development Bank. Glob. Policy.

[11] Harmer A., Buse K. (2014). The BRICS—A paradigm shift in global health?. Contemp. Politics.

[12] Gautier L., Harmer A., Tediosi F., Missoni E. (2014). Reforming the World Health Organization: What influence do the BRICS wield?. Contemp. Politics.

[13] Kickbusch I. (2014). BRICS’ contributions to the global health agenda. Bull. World Health Organ..

[14] BRICS Information Centre BRICS Health Ministers’ Meeting: Beijing Declaration. http://www.brics.utoronto.ca/docs/110711-health.html.

[15] Watt N.F., Gomez E.J., McKee M. (2014). Global health in foreign policy--and foreign policy in health? Evidence from the BRICS. Health Policy Plan..

[16] Marten R., McIntyre D., Travassos C., Shishkin S., Longde W., Reddy S., Vega J. (2014). An assessment of progress towards universal health coverage in Brazil, Russia, India, China, and South Africa (BRICS). Lancet.

[17] BRICS Information Centre Joint Communiqué of the BRICS Member States on Health. http://www.brics.utoronto.ca/docs/120522-health.html.

[18] BRICS Information Centre Compliance Reports. http://www.brics.utoronto.ca/compliance/index.html.

[19] Dirlikov E. (2015). BRICS health and tuberculosis control collaborations during an era of global health. Med. Anthropol. Theory.

[20] BRICS Information Centre Joint Communiqué of the BRICS Member States on Health on the Sidelines of the 67th WHA. http://www.brics.utoronto.ca/docs/140520-health.html.

[21] UN (2015). Resolution adopted by the General Assembly on 25 September 2015. A/RES/70/1. https://www.un.org/en/development/desa/population/migration/generalassembly/docs/globalcompact/A_RES_70_1_E.pdf.

[22] BRICS Information Centre Joint Communiqué of the BRICS Member States on Health on the Sidelines of the 69th World Health Assembly. http://www.brics.utoronto.ca/docs/160524-health.html.

[23] Weiß A. (2017). Soziologie Globaler Ungleichheiten.

[24] WHO (2018). World Health Statistics 2018. Monitoring Health for the SDGs.

[25] WHO (2011). Global Atlas on Cardiovascular Disease Prevention and Control.

[26] WHO (2014). Global Status Report on Noncommunicable Diseases 2014.

[27] Bovet P., Paccaud F. (2011). Cardiovascular Disease and the Changing Face of Global Public Health: A Focus on Low and Middle Income Countries. Public Health Rev..

[28] Di Cesare M., Khang Y.H., Asaria P., Blakely T., Cowan M.J., Farzadfar F., Guerrero R., Ikeda N., Kyobutungi C., Msyamboza K.P. (2013). Inequalities in non-communicable diseases and effective responses. Lancet.

[29] Callender T., Woodward M., Roth G., Farzadfar F., Lemarie J.C., Gicquel S., Atherton J., Rahimzadeh S., Ghaziani M., Shaikh M. (2014). Heart failure care in low- and middle-income countries: A systematic review and meta-analysis. PLoS Med..

[30] Dokainish H., Teo K., Zhu J., Roy A., AlHabib K.F., ElSayed A., Palileo-Villaneuva L., Lopez-Jaramillo P., Karaye K., Yusoff K. (2017). Global mortality variations in patients with heart failure: Results from the International Congestive Heart Failure (INTER-CHF) prospective cohort study. Lancet Glob. Health.

[31] Yancy C.W., Jessup M., Bozkurt B., Butler J., Casey D.E., Drazner M.H., Fonarow G.C., Geraci S.A., Horwich T., Januzzi J.L. (2013). 2013 ACCF/AHA guideline for the management of heart failure: Executive summary: A report of the American College of Cardiology Foundation/American Heart Association Task Force on practice guidelines. Circulation.

[32] Meyer J.W., Ramirez F.O., Soysal Y.N. (1992). World Expansion of Mass Education, 1870–1980. Sociol. Educ..

[33] Finnemore M. (1993). International organizations as teachers of norms: The United Nations Educational, Scientific, and Cutural Organization and Science Policy. Int. Organ..

[34] Frank D.J., Schofer E., Tuma N., Meyer J., Boli J., Thomas G.M. (1999). The Rationalization and Organization of Nature in the World Culture. Constructing World Culture: International Nongovernmental Organizations Since 1875.

[35] Meyer J.W., Rowan B. (1977). Institutionalized Organizations—Formal-Structure as Myth and Ceremony. Am. J. Sociol..

[36] Leisering L., Liu T., ten Brink T. (2017). Synthesizing disparate ideas: How a Chinese model of social assistance was forged. Glob. Soc. Policy.

[37] Liu T., Sun L. (2016). Urban social assistance in China: Transnational diffusion and national interpretation. J. Curr. Chin. Aff..

[38] Levitt P., Merry S. (2009). Vernacularization on the ground: Local uses of global women’s rights in Peru, China, India and the United States. Glob. Netw..

[39] Inoue K., Drori G.S. (2006). The Global Institutionalization of Health as a Social Concern: Organizational and Discursive Trends. Int. Sociol..

[40] Lakoff A. (2010). Two Regimes of Global Health. Humanity.

[41] Bramlage P., Bohm M., Volpe M., Khan B.V., Paar W.D., Tebbe U., Thoenes M. (2010). A global perspective on blood pressure treatment and control in a referred cohort of hypertensive patients. J. Clin. Hypertens..

[42] Stollberg H. (2009). Das medizinische System. Überlegungen zu einem von der Soziologie vernachlässigten Funktionssystem. Soz. Syst..

[43] Stein T. (2013). Medizin ohne Grenzen: Die Globalisierungsmechanismen der Schulmedizin. https://pub.uni-bielefeld.de/record/2638155.

[44] Yancy C.W., Jessup M., Bozkurt B., Butler J., Casey D.E., Colvin M.M., Drazner M.H., Filippatos G., Fonarow G.C., Givertz M.M. (2016). 2016 ACC/AHA/HFSA Focused Update on New Pharmacological Therapy for Heart Failure: An Update of the 2013 ACCF/AHA Guideline for the Management of Heart Failure: A Report of the American College of Cardiology/American Heart Association Task Force on Clinical Practice Guidelines and the Heart Failure Society of America. J. Am. Coll. Cardiol..

[45] Yancy C.W., Jessup M., Bozkurt B., Butler J., Casey D.E., Colvin M.M., Drazner M.H., Filippatos G.S., Fonarow G.C., Givertz M.M. (2017). 2017 ACC/AHA/HFSA Focused Update of the 2013 ACCF/AHA Guideline for the Management of Heart Failure: A Report of the American College of Cardiology/American Heart Association Task Force on Clinical Practice Guidelines and the Heart Failure Society of America. Circulation.

[46] Rohde L.E.P., Montera M.W., Bocchi E.A., Clausell N.O., Albuquerque D.C.d., Rassi S., Colafranceschi A.S., Freitas Junior A.F.d., Ferraz A.S., Biolo A. (2018). Diretriz Brasileira de Insuficiência Cardíaca Crônica e Aguda. Arq. Bras. Cardiol..

[47] Mareev V.Y., Fomin I.V., Ageev F.T., Begrambekova Y.L., Vasyuk Y.A., Garganeeva A.A., Gendlin G.E., Glezer M.G., Gautier S.V., Dovzhenko T.V. (2018). Russian Heart Failure Society, Russian Society of Cardiology. Russian Scientific Medical Society of Internal Medicine Guidelines for Heart failure: Chronic (CHF) and acute decompensated (ADHF). Diagnosis, prevention and treatment (In Russ.). Kardiologiia.

[48] Guha S., Harikrishnan S., Ray S., Sethi R., Ramakrishnan S., Banerjee S., Bahl V.K., Goswami K.C., Banerjee A.K., Shanmugasundaram S. (2018). CSI position statement on management of heart failure in India. Indian Heart J..

[49] Heart Failure Group of Chinese Society of Cardiology of Chinese Medical Association, Chinese Heart Failure Association of Chinese Medical Doctor Association, Editorial Board of Chinese Journal of Cardiology (2018). Chinese Heart Failure Diagnosis and Treatment Guideline 2018. Chin. J. Card..

[50] Corbin J.M., Strauss A.L. (2008). Basics of Qualitative Research: Techniques and Procedures for Developing Grounded Theory.

[51] Mueller C., Camm A.J., Lüscher T.F., Maurer G., Serruys P.W. (2018). Chronic heart failure diagnosis: Biomarkers. ESC CardioMed.

[52] Van der Meer P., Gaggin H.K., Dec G.W. (2019). ACC/AHA Versus ESC Guidelines on Heart Failure: JACC Guideline Comparison. J. Am. Coll. Cardiol..

[53] McMurray J.J.V., Packer M., Desai A.S., Gong J., Lefkowitz M.P., Rizkala A.R., Rouleau J.L., Shi V.C., Solomon S.D., Swedberg K. (2014). Angiotensin–Neprilysin Inhibition versus Enalapril in Heart Failure. N. Engl. J. Med..

[54] Anonymous co-author of the 2016 ESC Guidelines (2019). Personal communication.

[55] Anonymous co-author of the ACCF/AHA Guideline (2019). Personal communication.

[56] Normand C., Linde C., Singh J., Dickstein K. (2018). Indications for Cardiac Resynchronization Therapy: A Comparison of the Major International Guidelines. JACC Heart Fail..

[57] Timmermans S., Berg M. (2003). The Gold Standard: The Challenge of Evidence-Based Medicine and Standardization in Health Care.

[58] Weisz G., Cambrosio A., Keating P., Knaapen L., Schlich T., Tournay V.J. (2007). The emergence of clinical practice guidelines. Milbank Q..

[59] Konstam M., Dracup K., Baker D. (1994). Heart Failure: Evaluation and Care of Patients with Left-Ventricular Systolic Dysfunction. AHCPR Clinical Practice Guidelines, No. 11.

[60] ESC Task Force on Heart Failure (1995). Guidelines for the diagnosis of heart failure. Eur. Heart J..

[61] Williams J.F., Bristow M.R., Fowler M.B., Francis G.S., Garson A. (1995). Guidelines for the evaluation and management of heart failure. Report of the American College of Cardiology/American Heart Association Task Force on Practice Guidelines (Committee on Evaluation and Management of Heart Failure). J. Am. Coll. Cardiol..

[62] Antman E.M., Bax J., Chazal R.A., Creager M.A., Filippatos G., Halperin J.L., Houser S., Lindenfeld J., Pinto F.J., Vardas P. (2016). Updated Clinical Practice Guidelines on Heart Failure: An International Alignment. J. Am. Coll. Cardiol..

[63] Anonymous co-author of the 2016 ESC Guidelines (2019). Personal communication.

[64] Ezekowitz J.A., O’Meara E., McDonald M.A., Abrams H., Chan M., Ducharme A., Giannetti N., Grzeslo A., Hamilton P.G., Heckman G.A. (2017). 2017 Comprehensive Update of the Canadian Cardiovascular Society Guidelines for the Management of Heart Failure. Can. J. Card..

[65] Seferovic P.M., Ponikowski P., Anker S.D., Bauersachs J., Chioncel O., Cleland J.G.F., de Boer R.A., Drexel H., Ben Gal T., Hill L. (2019). Clinical practice update on heart failure 2019: Pharmacotherapy, procedures, devices and patient management. An expert consensus meeting report of the Heart Failure Association of the European Society of Cardiology. Eur. J. Heart Fail..

[66] De Souza Queiroz L.R., Jadelson A. (2013). Sociedade Brasileira de Cardiologia—70 anos—Uma História de Sucesso—1943/2013.

[67] Sociedade Brasileira de Cardiologia (1992). Consenso Brasileiro para o Tratamento da Insuficiência Cardíaca. Arq. Bras. Cardiol..

[68] Ponikowski P., Voors A.A., Anker S.D., Bueno H., Cleland J.G.F., Coats A.J.S., Falk V., González-Juanatey J.R., Harjola V.P., Jankowska E.A. (2017). ESC Guidelines for the Diagnosis and Treatment of Acute and Chronic Heart Failure (In Russ.). Rus. J. Card..

[69] Heart Failure Group of Chinese Society of Cardiology, Editorial Board of Chinese Journal of Cardiology (2014). Chinese Heart Failure Diagnosis and Treatment Guideline 2014. Chin. J. Card..

[70] Anonymous co-author of the Chinese HF Guideline 2018 (2019). Personal communication.

[71] Anonymous co-author of the Chinese HF Guideline 2018 (2019). Personal communication.

[72] South African Medical Association (1998). Heart failure clinical guideline. S. Afr. Med. J..

[73] SA Heart® Guidelines. https://www.saheart.org/cms-home/category/17.

[74] Mpe M.T., Klug E.Q., Silwa K.S., Hitzeroth J., Smith D.A. (2013). Heart Failure Society of South Africa (HeFSSA) perspective on the European Society of Cardiology (ESC) 2012 chronic heart failure guideline. S. Afr. Med. J..

[75] Tregenna F., Tsela M. (2012). Inequality in South Africa: The distribution of income, expenditure and earnings. Dev. S. Afr..

[76] Ataguba J.E., McIntyre D. (2012). Paying for and receiving benefits from health services in South Africa: Is the health system equitable?. Health Policy Plan..

[77] Stewart S., Wilkinson D., Hansen C., Vaghela V., Mvungi R., McMurray J., Sliwa K. (2008). Predominance of Heart Failure in the Heart of Soweto Study Cohort. Circulation.

[78] Anonymous reviewer (2020). Comment on this article.

[79] Stamatakis E., Weiler R., Ioannidis J.P.A. (2013). Undue industry influences that distort healthcare research, strategy, expenditure and practice: A review. Eur. J. Clin. Investig..

[80] Kaushik S.R. (2017). Pharmocracy. Value, Politics & Knowledge in Global Biomedicine.

[81] Choudhry N.K., Stelfox H.T., Detsky A.S. (2002). Relationships Between Authors of Clinical Practice Guidelines and the Pharmaceutical Industry. JAMA.

[82] Norris S.L., Holmer H.K., Ogden L.A., Burda B.U. (2011). Conflict of Interest in Clinical Practice Guideline Development: A Systematic Review. PLoS ONE.

[83] Jakovljevic M., Potapchik E., Popovich L., Barik D., Getzen T.E. (2017). Evolving Health Expenditure Landscape of the BRICS Nations and Projections to 2025. Health Econ..

[84] Van Montfort C., Sun L., Zhao Y. (2018). Stability by change–the changing public-private mix in social welfare provision in China and the Netherlands. J. Chin. Govern..

[85] Gu E., Page-Jarrett I. (2018). The top-level design of social health insurance reforms in China: Towards universal coverage, improved benefit design, and smart payment methods. J. Chin. Govern..

